# Bolalipid-Doped Liposomes: Can Bolalipids Increase the Integrity of Liposomes Exposed to Gastrointestinal Fluids?

**DOI:** 10.3390/pharmaceutics11120646

**Published:** 2019-12-03

**Authors:** Sindy Müller, Kai Gruhle, Annette Meister, Gerd Hause, Simon Drescher

**Affiliations:** 1 Institute of Pharmacy, Biophysical Pharmacy, Martin Luther University (MLU) Halle-Wittenberg, 06120 Halle (Saale), Germany; sindy.mueller@pharmazie.uni-halle.de (S.M.); kai.gruhle@pharmazie.uni-halle.de (K.G.); 2 ZIK HALOmem and Institute of Biochemistry and Biotechnology, Charles Tanford Protein Center, MLU Halle-Wittenberg, 06120 Halle (Saale), Germany; annette.meister@chemie.uni-halle.de; 3 Biocenter, MLU Halle-Wittenberg, 06120 Halle (Saale), Germany; gerd.hause@biozentrum.uni-halle.de

**Keywords:** bolalipids, bolaamphiphiles, phospholipids, liposomes, bolasomes, in vitro stability, dithionite assay, calcein release assay, freeze-drying

## Abstract

The use of archaeal lipids and their artificial analogues, also known as bolalipids, represents a promising approach for the stabilization of classical lipid vesicles for oral application. In a previous study, we investigated the mixing behavior of three single-chain alkyl-branched bolalipids **PC-C32(1,32C*n*)-PC** (*n* = 3, 6, 9) with either saturated or unsaturated phosphatidyl-cholines. We proved, that the bolalipids **PC-C32(1,32C6)-PC** and **PC-C32(1,32C9)-PC** show miscibility with 1-palmitoyl-2-oleoyl-*sn*-glycero-3-phosphocholine (POPC) and 1,2-dioleoyl-*sn*-glycero-3-phosphocholine (DOPC). In the present work, we extended our vesicle system to natural lipid mixtures using phosphatidylcholine from soy beans, and we investigated the effect of incorporated bolalipids on the integrity of these mixed liposomes (bolasomes) in different gastrointestinal fluids using a dithionite assay and a calcein release assay in combination with particle size measurements. Finally, we also studied the retention of calcein within the bolasomes during freeze-drying. As a main result, we could show that in particular **PC-C32(1,32C6)-PC** is able to increase the stability of bolasomes in simulated gastric fluid—a prerequisite for the further use of liposomes as oral drug delivery vehicles.

## 1. Introduction

Liposomes were discovered in 1965 by Bangham and have become the most successful drug delivery system [1,2]. Since it is known that liposomes can increase the absorption of substances from the gastrointestinal tract (GIT)—shown by Sessa et al. in 1970 [3]—the idea for using liposomes for oral drug delivery purposes exists, and has been followed for decades by many research groups [4,5,6,7,8]. The encapsulation of drugs in liposomes has many advantages: In addition to the high encapsulation efficiency of lipophilic and hydrophilic drugs, as well as their high biocompatibility, liposomes protect their cargo from external influences such as enzymatic degradation or premature metabolism [9]. Furthermore, liposomal formulations of highly potent drugs also show fewer side effects than non-encapsulated substances [10,11,12]. The production of liposomes is relatively inexpensive, up-scalable, and the lipid composition of the liposomes can be easily adapted to the application of interest. Nevertheless, conventional liposomes show instabilities in the human GIT. In particular, acidic conditions, the influence of bile salts, and enzymes usually lead to a destabilization of classical phospholipid liposomes, and thus the leakage of encapsulated drugs [13,14,15,16,17,18]. On the one hand, it is therefore necessary to stabilize liposomes in order to use them for oral administration. For this purpose, various approaches were evolved in recent years including the variation of the lipid composition using phospholipids with high phase transitions and/or cholesterol [15,19,20], surface coating with polymers, proteins and chitosan [4,6,7,21,22,23,24,25], or the incorporation of bile salts into the lipid bilayer [5,26,27,28,29,30]. On the other hand, the goal of every drug delivery system is to provide its cargo at a therapeutic concentration for a defined period of time to a particular site of action. Over-stabilized nanoparticles could possibly leave the GIT unchanged, without releasing the encapsulated drug. Thus, it is important to find a compromise between the stability of modified liposomes and the effective protection of their cargo, as well as a timely release of the loaded substances. 

One way to stabilize liposomes is the use of archaeal membrane lipids. Archaea, representing the third domain of life, proliferate under extreme living conditions due to their outstanding stability of the cell membrane. The tetraether lipids (TELs) of certain species of Archaea, also named bolalipids or bolaamphiphiles [31], contribute in particular to the stabilization of the membranes against high salt concentrations, low pH values, and high temperatures. In recent years, these stabilizing properties have made TELs very interesting for the modification of lipid formulations, especially liposomes. Lipid vesicles containing archaeal lipids are called archaeosomes, and they have some desirable properties for use in drug delivery systems: They show a low permeability to small ions, which is important especially for oral application regarding the acidic environment of the stomach [32,33,34], and they can significantly improve the bioavailability of encapsulated drugs after oral administration [35,36,37]. Archaeosomes also show an increased stability against bile salts when compared to conventional liposomes, which is presumably due to the presence of TELs preventing the incorporation of bile salts into the lipid membrane [38,39]. Furthermore, archaeosomes can be sterilized by autoclaving without significant alterations in vesicle diameter, particle size distribution, or fusion of liposomes [40]. And finally, archaeal lipids are non-toxic upon administration by both intravenous and oral routes [41]. But, since the full synthesis of TELs—firstly described by Kakinuma [42]—and also the extraction of archaeal lipids from natural sources is expensive and time consuming [43,44], the search for alternative chemical structures is still indispensable. 

**Figure 1:**
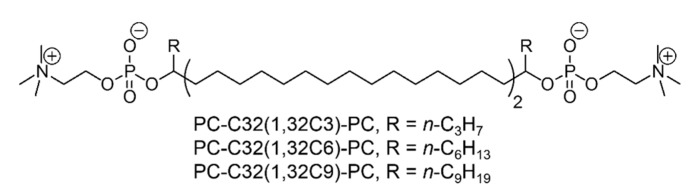
Chemical structure of single-chain, alkyl-branched bolalipids **PC-C32(1,32C*n*)-PC** with *n* = 3, 6, 9 [52]. The bolalipids **PC-C32(1,32C6)-PC** and **PC-C32(1,32C9)-PC** are used in this study.

During the last decade, we synthesized and characterized a large set of archaeal model lipids, that are simplified analogues of TELs found in Archaea. But, it still remains challenging to keep the chemical structure of these bolalipids as simple as possible by maintaining their stabilizing properties. One of the simplest bolalipid, the **PC-C32-PC**, consists of two phosphocholine (PC) headgroups connected by an unmodified C32 alkyl chain [45]. This model lipid shows no tendency to be incorporated into lipid membranes composed of 1,2-dipalmitoyl-*sn*-glycero-3-phosphocholine (DPPC) and 1-palmitoyl-2-oleoyl-*sn*-glycero-3-phosphocholine (POPC), respectively [46]. The reason therefore can be found in packing problems caused by the large space requirement of the PC headgroups compared to the small cross-sectional area of the single alkyl chain. As a result, void volume is created when **PC-C32-PC** is inserted into a stretched conformation in the lipid bilayer. This energetically unfavorable situation leads to the separation of **PC-C32-PC** and phospholipid, that is DPPC or POPC [46]. One possibility to overcome this packing problem is the enlargement of the hydrophobic part of the bolalipid. Several attempts including, for example, the incorporation of a phenyl group into the alkyl chain of the bolalipid [47,48,49,50,51] have failed. Hence, we synthesized a set of single-chain bolalipids bearing a lateral alkyl chain of different length right next to the PC headgroups, the **PC-C32(1,32C*n*)-PC** with *n* = 3, 6, 9 [52] (Figure 1). 

In the case of lateral hexyl (C6) and nonyl (C9) alkyl chains, i.e., **PC-C32(1,32C6)-PC** and **PC-C32(1,32C9)-PC**, we could demonstrate that these groups are sufficient to fill the void volume in a mixed bolalipid/phospholipid membrane when unsaturated phospholipids, that is POPC or DOPC, are used [53]. As a result, we were able to produce for the first time storage-stable liposomes (bolasomes) composed of POPC and DOPC with 20 mol% of **PC-C32(1,32C6)-PC** and **PC-C32(1,32C9)-PC**, respectively. 

In the present work, we focused on stability studies of our bolasomes in different artificial digestion media, namely simulated gastric fluid (SGF) and simulated intestinal fluid (SIF). Besides **PC-C32(1,32C6)-PC**/DOPC (1/4, *n*/*n*) and **PC-C32(1,32C9)-PC**/DOPC (1/4, *n*/*n*) [53], we also used a natural phospholipid mixture, that is Phospholipon 90G^®^, containing at least 95% of phosphatidyl-cholines from soy beans (soyPC), for our stability measurements, since natural phospholipids have been attracting more and more attention in the pharmaceutical industry [18,54]. 

This study was divided into two parts. At first, we characterized the new bolasome mixture **PC-C32(1,32C9)-PC**/soyPC (1/4, *n*/*n*) using differential scanning calorimetry (DSC), transmission electron microscopy (TEM) of vitrified specimens (cryo-TEM), as well as replica of freeze-fractured samples (FFEM), and dynamic light scattering (DLS). In the second part of the paper, we investigated the integrity of our bolasomes in phosphate buffered saline (PBS), and two digestion media, that are SGF and SIF. For the permeability test of the bolasomes against small ions, we used the dithionite assay. The stability of our bolasomes in artificial gastrointestinal fluids was checked by the calcein release assay and DLS. Finally, we investigated the retention of the fluorescent dye calcein after freeze-drying and rehydration, using trehalose and lysine as cryoprotectants, respectively. 

## 2. Materials and Methods 

### 2.1. Materials 

The bolalipids (1*RS*,32*RS*)-1,32-dihexyldotriacontane-1,32-diylbis[2-(trimethylammonio)ethyl phosphate] (**PC-C32(1,32C6)-PC**) and (1*RS*,32*RS*)-1,32-dinonyldotriacontane-1,32-diylbis[2-(tri-methylammonio)ethyl phosphate] (**PC-C32(1,32C9)-PC**) were synthesized according to procedures described previously [52]. DOPC and 1,2-dipalmitoyl-*sn*-glycero-3-phosphoethanolamine-*N*-(7-nitro-2,1,3-benzoxadiazol-4-yl) (ammonium salt) (NBD-PE) were purchased from Avanti Polar Lipids (Alabaster, AL, USA) and used without further purification. SoyPC (Phospholipon 90 G^®^) was kindly provided by Lipoid GmbH (Ludwigshafen, Germany). Buffer salts were obtained from Sigma Aldrich Co. (Steinheim, Germany). Calcein disodium salt was purchased from Fluka Chemie GmbH (Buchs, Switzerland). Simulated gastric fluid (SGF) was prepared by adding pepsin at a concentration of 1 mg/mL to a buffer (7.57 mM citric acid, 8.2 mM HCl, 119.78 mM NaCl and 2.7 mM KCl) at pH = 1, directly before use. Simulated intestinal fluid (SIF) was prepared by adding sodium taurocholate and trypsin, in a concentration of 5.9 mg/mL and 1.0 mg/mL, respectively, to a phosphate buffer at pH = 8. 

### 2.2. Methods 

#### 2.2.1. Sample Preparation 

Vesicles were prepared by the film method firstly described by Bangham [55]. Briefly, binary lipid mixtures were prepared by mixing appropriate volumes of the lipid stock solutions in chloroform/methanol (2/1, *v*/*v*). After evaporating the organic solvent in a stream of nitrogen, the lipid films obtained were stored in an evacuated flask for at least 24 h in order to eliminate all traces of the solvent. The lipid dispersions were prepared by adding a certain volume of the buffer to obtain the correct lipid concentration. Afterwards, the lipid samples were vortexed for 5 min at 70 °C to obtain a homogenous suspension. To get mostly uniform liposomes (bolasomes), lipid dispersions were extruded 31 times through a polycarbonate membrane of 100 nm pore size at a temperature roughly 10 K above the transition temperature observed in the DSC experiment. All lipid dispersions were stored at 4 °C in a glass vial filled with nitrogen to prevent oxidation of the lipids. 

#### 2.2.2. Differential Scanning Calorimetry (DSC) 

As published previously [53,56], DSC measurements were carried out using a MicroCal VP-DSC differential scanning calorimeter (MicroCal Inc. Northampton, MA, USA). Before each measurement, the lipid dispersion (*c* = 3 mM) and the reference were degassed under vacuum while stirring. For the measurements, a temperature interval of 5 to 80 °C and a heating rate of 60 K/h were used. To verify reproducibility, three consecutive scans were recorded for each lipid sample. Finally, the buffer-buffer baseline was subtracted from the thermogram of the sample. All DSC scans were evaluated using MicroCal Origin 8.0 software (OriginLab Corporation, Northampton, MA, USA). 

#### 2.2.3. Transmission Electron Microscopy of Vitrified Specimens (Cryo-TEM) 

In accordance to a procedure described previously [56], vitrified specimens for cryo-TEM were prepared by a blotting procedure using an EM GP grid plunger (Leica, Wetzlar, Germany), and an atmospheric chamber with controlled humidity (80%) and temperature (*T* = 22 °C). For cryo-fixation, a drop of the sample solution (*c* = 3 mM) was placed onto an EM grid that is coated with a holey carbon film (C-flat, Protochip Inc., Raleight, NC, USA). After the excess solution was removed with a filter paper, vitrification of the thin film was achieved by rapid plunging of the grid into liquid ethane. The vitrified specimens were kept at temperatures below 108 K during storage, transfer to the microscope, and investigation. Specimens were examined with a Libra 120 Plus TEM (Carl Zeiss Microscopy GmbH, Jena, Germany) operating at 120 kV The microscope was equipped with a Gatan 626 cryotransfer system and with a BM-2k-120 Dual-Speed on axis SSCCD-camera (TRS, Moorenweis, Germany). 

#### 2.2.4. Transmission Electron Microscopy of Replica of Freeze-Fractured Samples (FFEM) 

As published previously [53], the lipid samples (*c* = 6 mM) were cryo-fixed using a propane jet-freeze device JFD 030 (BAL-TEC, Balzers, Lichtenstein). Afterwards, the samples were freeze-fractured at −150 °C without etching, using a freeze fracture/freeze etching system BAF 060 (BAL-TEC, Balzers, Liechtenstein). The surfaces were firstly shadowed with platinum (2 nm layer, shadowing angle 45°) and secondly with carbon (20 nm layer, shadowing angle 90°). The replica were first floated in sodium chloride (4%, Roth, Karlsruhe, Germany) for 30 min, then rinsed in distilled water (10 min), washed in 30% acetone (Roth, Karlsruhe, Germany) for 30 min, and rinsed again in distilled water (10 min). The replica were mounted on copper grids, coated with Formvar film and investigated using a transmission electron microscope (LIBRA 120 PLUS, Carl Zeiss Microscopy GmbH, Jena, Germany), operating at 120 kV. Images were taken with a BM-2k-120 Dual-Speed on an axis SSCCD-camera (TRS, Moorenweis, Germany). 

#### 2.2.5. Dynamic Light Scattering (DLS) 

DLS experiments were carried out with a Litesizer 500 (Anton Paar GmbH, Graz, Austria). A 3 mW laser with a wavelength of *λ* = 658 nm and back scattering angle was used. All samples were measured in quartz ultramicro-cuvettes (path length 10 mm, Hellma Analystics, Müllheim, Ger-many). Extruded lipid dispersions containing liposomes with a lipid concentration of 3 mM in case of stability experiments and 10 mM in the case of in vitro experiments were used, respectively. For in vitro experiments, samples were diluted 1:10 with either PBS, SGF, or SIF and incubated at 37 °C for 4 h in the device during measurement. Each particle size measurement consisted of three separate runs with a measuring time of 6 × 10 s each. The experimental data were further analyzed with the aid of OriginPro 8 software (OriginLab Corporation, Northampton, MA, USA). 

#### 2.2.6. Dithionite Fluorescence Assay 

The permeability of liposomes to small ions was tested by the dithionite assay described by McIntyre et al. [57]. We used two sets of lipid samples: the first with 1 mol% of NBD-PE, the second without NBD-PE. Both sets of liposomes were prepared as described above using a total lipid concentration of 0.167 mM. The fluorescence measurements were carried out on a Fluoromax 2 (Horiba Scientific, Kyoto, Japan) with constant stirring, and a total sample volume of 800 µL. The sample cuvette (path length 10 mm, Hellma Analytics, Müllheim, Germany) was held at a constant temperature using a water bath. The time-dependent measurement was carried out with a total measuring time of 6 h, a time increment of 5 min, and an integration time of 10 s for each incremental time point. The excitation wavelength was 463 nm; the emission was detected at 530 nm. The measurement was carried out in “Anti-Photo-Bleach” mode. Before the addition of excess dithionite to the lipid sample, the initial 15 min of steady state fluorescence was averaged (per three runs), representing the value that corresponded to 100% of unreacted NBD lipids (*F_init_*). The addition of excess dithionite resulted in the quick drop of the fluorescence until all NBD lipids in the outer leaflet were reduced. After this, we attributed any subsequent decay of the fluorescence (*F_dith_*) to the permeation of the dithionite ions through the lipid bilayer and the reaction with the NBD molecules in the inner leaflet. After 10 h, all vesicles were destroyed using Triton X-100 (10%, *v*/*v*) to determine the fluorescence value of the sample when all NBD lipids were reduced (*F_trit_*). The relative fluorescence corresponded to:

(1) $$relative\;fluorescence=\frac{F_{dith}-F_{trit}}{F_{init}}.$$

The time of addition of dithionite was set to *t* = 0 min; the value of fluorescence before the addition of dithionite was normalized to 1. The fluorescence of the reference liposomes was subtracted from all fluorescence measurements of the NBD-labelled liposomes. The results were evaluated using MicroCal Origin 8.0 software (OriginLab Corporation, Northampton, MA, USA). 

#### 2.2.7. Calcein Release Assay 

Lipid films were hydrated with PBS (pH = 7.4) containing 30 mM calcein to achieve a total lipid concentration of 10 mM. Liposomes were sonicated 2 × 5 min, with a 5 min break. The non-encapsulated calcein was separated by size exclusion chromatography (SEC) using PD-10 Desalting Columns containing a Sephadex^®^ G-25 medium (GE Healthcare, Milwaukee, WI, USA). The release of calcein was determined at 37 °C using a POLARstar Omega (BMG Labtech, Ortenberg, Germany) after the injection of the liposomes in PBS (pH = 7.4), SGF (pH = 1.0), or SIF (pH = 8.0) resulting in a 1:10 dilution of the dispersions. The fluorescence was measured at 485 nm excitation and 520 nm emission wavelength. Due to the fact that the fluorescence of calcein is pH-dependent, and to be further able to compare the results of different measurements, calibration curves of different calcein concentrations at the three different pH values were used to convert the measured fluorescence intensities of all lipid samples to the intensity at pH 7.4 [58]. The emission of liposomes without calcein was used as a negative control. The emission of the samples after the destruction of the liposomes using Triton X-100 (10%, *v*/*v*) was set as 100 % release. All measurements were performed in triplicate in 96 well plates (Greiner Bio-One, Frickenhausen, Germany). The leakage of calcein over time was calculated as follows:

(2) $$calcein\;release\;\left(\%\right)=\frac{FE-FE_{0}}{FE_{Trit}-FE_{0,Trit}}\times 100\%,$$

where *FE* is the fluorescence intensity at a certain time, *FE*_0_ is the fluorescence intensity of the liposomes without calcein, *FE_Trit_* is the fluorescence intensity of the calcein-liposomes after destruction with Triton X-100 (10 %, *v*/*v*), and *FE*_0,*Trit*_ is the fluorescence intensity of the liposomes without calcein after the destruction with Triton X-100 (10%, *v*/*v*). The experimental data were analyzed using OriginPro 8 software (OriginLab Corporation, Northampton, MA, USA). 

#### 2.2.8. Freeze-Drying and Calcein Retention 

The liposomal suspensions were prepared as described above by adding a certain volume of PBS (pH = 7.4), containing 30 mM calcein and 40 mM lysine or 300 mM trehalose, to the lipid films to obtain a total lipid concentration of 10 mM. The non-encapsulated calcein was separated from liposomes by SEC using PD-10 Desalting Columns containing a Sephadex^®^ G-25 medium (GE Healthcare, Milwaukee, USA), with PBS as eluent containing 40 mM lysine or 300 mM trehalose. A 1 mL aliquot of the liposomal dispersion was pre-frozen at −25 °C for 30 min followed by drying, which was carried out at −50 °C for 24 h using a freeze-dryer Heto CT60 (Heto-Holten A/S, Allerød, Denmark). Afterwards, the dried liposomes were rehydrated with MilliQ water, to their original volume. The percentage of calcein retained in the liposomes after freeze-drying and rehydration was determined at 25 °C by measuring the fluorescence intensity of calcein at 485 nm excitation and 520 nm emission wavelength using a POLARstar Omega (BMG Labtech, Ortenberg, Germany). All measurements were performed in triplicate in 96 well plates (Greiner Bio-One, Frickenhausen, Germany). The retention (%) was calculated according to the following equation proposed by Crowe et al. [59]

(3) $$retention\;\left(\%\right)=\frac{\left(F_{a}^{\prime }-F_{b}^{\prime }\right)/F_{a}^{\prime }}{\left(F_{a}-F_{b}\right)/F_{a}}\times 100\%,$$

where $F_{b}$ is the fluorescence intensity before freeze-drying, $F_{a}$ is the fluorescence intensity before freeze-drying but after destruction of the liposomes with Triton X, $F_{b}^{\prime }$ is the fluorescence intensity after freeze-drying and rehydration, and $F_{a}^{\prime }$ is the fluorescence intensity after freeze-drying and rehydration, and also after the destruction of the liposomes with Triton X-100 (10%, *v*/*v*). 

The encapsulation efficiency of the liposomes of calcein before freeze-drying was calculated according to the following equation

(4) $$encapsulation\;efficiency\;\left(\%\right)=\frac{F_{Trit}-F_{i}}{F_{Trit}}\times 100\%,$$

where $F_{i}$ is the fluorescence intensity of the lipid sample measured before the freeze-drying and $F_{Trit}$ is the fluorescence intensity of the lipid sample after the destruction of the liposomes using Triton X-100 before the freeze-drying, respectively.

## 3. Results and Discussion 

In a previous publication, we showed that the two single-chain alkyl-branched bolalipids **PC-C32(1,32C6)-PC** and **PC-C32(1,32C9)-PC**—bearing a lateral C6 or C9 alkyl chain right next to the PC headgroups (see Figure 1)—can be incorporated into lipid vesicles composed of unsaturated phos-pholipids, POPC and DOPC, respectively [53]. This leads to the formation of mixed bolalipid/ phospholipid vesicles (bolasomes). The miscibility of both lipid components was proved by DSC and EM investigations. In the case **PC-C32(1,32C9)-PC**, these bolasomes are stable in storage at 4 °C for at least three weeks, which was checked by DLS measurements. Since the miscibility between bolalipid and POPC/DOPC increases with the length of the bolalipid’s lateral alkyl chains, **PC-C32(1,32C9)-PC** is recommended for further studies [53]. Hence, we used this bolalipid for miscibility studies with phosphatidylcholines derived from soy beans (soyPC).

### 3.1. Miscibility of PC-C32(1,32C9)-PC with Phosphatidylcholines Derived from Soy Beans (soyPC) 

In addition to the pure synthetic phospholipids POPC and DOPC, we used a natural phospho-lipid mixture, Phospholipon 90G^®^, which contains at least 95% of phosphatidylcholines from soy beans (soyPC). In accordance to our previous study [53], we used 20 mol% of bolalipid in our mixed bolasomes. 

#### 3.1.1. DSC Measurements 

**Figure 2:**
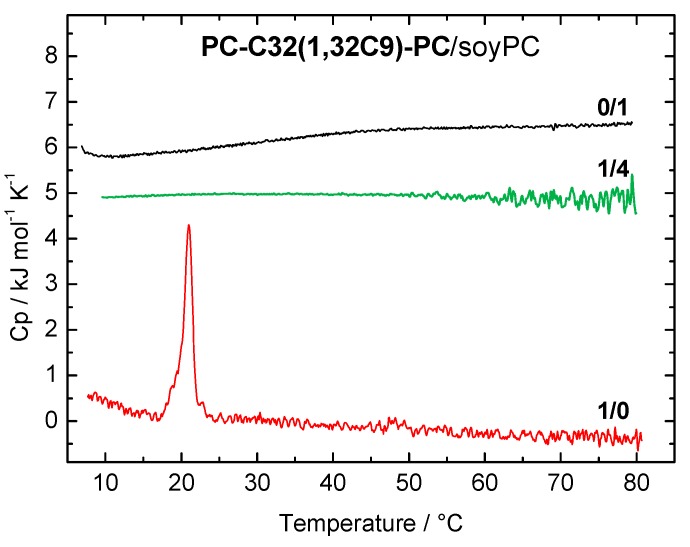
Differential scanning calorimetry (DSC) heating curve of **PC-C32(1,32C9)-PC**/soyPC mixture (1/4, *n*/*n*; *c* = 3 mM in PBS at pH = 7.4; green line). DSC heating curves of pure bolalipid (red line) and soyPC (black line) are shown for comparison. The curves are shifted vertically for clarity.

The DSC heating curves of the **PC-C32(1,32C9)-PC**/soyPC mixture (1/4, *n*/*n*; green line) and both pure components **PC-C32(1,32C9)-PC** (red line) and soyPC (black line), respectively, are shown in Figure 2. Pure soyPC showed no phase transition in the temperature range investigated (5–80 °C), which is due to the fact that the main transition temperature (*T*_m_) of soyPC is below 0 °C. Depending on the fatty acid composition of natural phospholipids, *T*_m_ can vary between −50 and −20 °C [60]. The bolalipid **PC-C32(1,32C9)-PC** shows a transition at *T*_m_ = 20.8 °C and it self-assembles into fibers below and sheet-like aggregates above this *T*_m_ [52]. In the 1/4 mixture of **PC-C32(1,32C9)-PC** and soyPC (Figure 2, green line), no transition can be observed in the DSC heating curve. This indicates a good miscibility between both components, which is also found for **PC-C32(1,32C9)-PC**/POPC and /DOPC mixtures, respectively [53]. 

For the further investigations regarding the morphology of the mixed **PC-C32(1,32C9)-PC**/ soyPC bolasomes, we extruded this lipid mixture through a polycarbonate membrane with a 100 nm pore size, and visualized the aggregates formed using electron microscopy (EM). 

#### 3.1.2. Cryo-TEM and FFEM Investigations 

**Figure 3:**
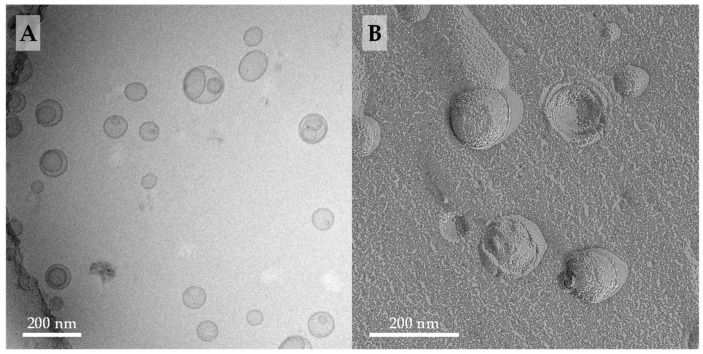
(**A**) Cryo-TEM image (*c* = 3 mM) and (**B**) EM image of freeze-fractured replica (FFEM) (*c* = 6 mM) of an aqueous suspension of **PC-C32(1,32C9)-PC**/soyPC mixture (1/4, *n*/*n*; in PBS pH = 7.4). The lipid sample was extruded through a 100 nm membrane.

Cryo-TEM is considered the gold standard for the visualization of lipid vesicles, since the shape and structures of these vesicles are preserved very close to their native state [61]. When compared to TEM of stained samples, neither contrasting agents nor drying procedures are needed for cryo-TEM during sample preparation—both could lead to artefacts [62]. We therefore prepared cryo-TEM and FFEM samples of a 1/4 mixture of **PC-C32(1,32C9)-PC**/soyPC in PBS at pH = 7.4. The results are shown in Figure 3. 

The cryo-TEM image of extruded **PC-C32(1,32C9)-PC**/soyPC mixture (1/4, *n*/*n*) revealed the presence of many unilamellar vesicles with diameters between 100–150 nm (Figure 3A). Additionally, some oligolamellar and also multivesicular vesicles could be found. When compared to the cryo-TEM images of the corresponding POPC or DOPC mixtures published previously [53], we noticed two differences: first, the POPC- as well as DOPC-based bolasomes are somewhat larger in size, up to diameters of 200 nm, and second, in mixtures with DOPC, a small number of elongated liposomes could be observed, which were not present in the soyPC mixture. Since all samples were prepared under the same conditions, both effects could be attributed to soyPC. This mixture of phosphati-dylcholines, derived from a natural source, includes lipids with different alkyl chains lengths and a variable degree of saturation. Both can significantly affect the formation of lipid vesicles [54,63]. 

With the aid of FFEM, the morphology of fractured vesicles could be investigated in more detail. EM images of a replica of a freeze-fractured **PC-C32(1,32C9)-PC**/soyPC mixture (1/4, *n*/*n*) showed the presence of vesicles with diameters ranging from 80–150 nm (Figure 3B). These EM images are com-parable to FFEM images of the corresponding **PC-C32(1,32C6)-PC**/DOPC and **PC-C32(1,32C9)-PC**/ DOPC mixtures published previously [53] and, hence, we concluded that **PC-C32(1,32C9)-PC** is miscible with soyPC—a fact, which was also confirmed by DSC measurements described above. Moreover, the bolalipid is arranged in a membrane-spanning fashion within the soyPC bilayer since no inner fracture faces could be observed in the FFEM images of the bolalipid/phospholipid mixtures investigated in this study and also the previous work [53]. 

It is noteworthy that the bolasomes of **PC-C32(1,32C9)-PC**/soyPC had a relative rough surface (Figure 3B), which is, however, not as pronounced as in the **PC-C32(1,32C6)-PC**/DOPC and the **PC-C32(1,32C9)-PC**/DOPC mixtures [53]. This roughness is probably attributed to the formation of bolalipid complexes within the mixed vesicles; a fact, which is in accordance to investigations done by Beveridge et al. [64] using archaeosomes with an increasing amount of membrane-spanning TELs. 

#### 3.1.3. DLS Measurements 

In order to investigate the storage stability of the mixed **PC-C32(1,32C9)-PC**/soyPC vesicles, we measured their particle size (z-average) and their particle size distribution (polydispersity index, PdI) right after extrusion (day 0) and after different times of storage at 4 °C (day 3 and day 7). 

**Figure 4:**
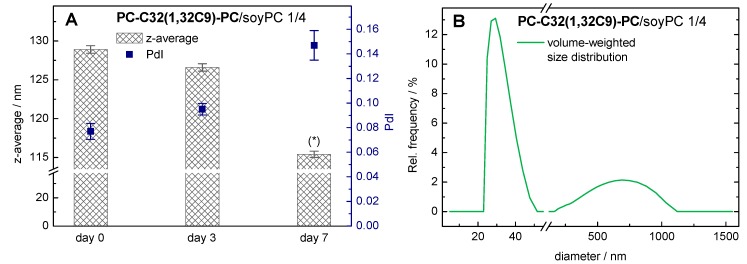
Data from dynamic light scattering (DLS) measurements: influence of time of storage at 4 °C on the particle size. (**A**) Mean and s.d. (*n* = 3) of z-average (bars) and polydispersity index PdI (squares) of a 1/4 **PC-C32(1,32C9)-PC**/soyPC mixture (*c* = 3 mM in PBS at pH = 7.4) are shown (* data should be taken with caution; see text for further explanation). (**B**) Volume-weighted particle size distribution of the same bolasomes after seven days of storage at 4 °C.

With the use of DLS, the autocorrelation function obtained can be analyzed using the cumulant fit, from which we can get the hydrodynamic diameter (z-average) and the PdI for the vesicular aggregates. For the **PC-C32(1,32C9)-PC**/soyPC 1/4 mixture at day 0, a z-average of 129 nm and a PdI of 0.08 were obtained (Figure 4A). 

After seven days of storage at 4 °C, the z-average decreased to 115 nm, whereas the PdI increased to 0.15. Since only PdI-values below 0.1 can be assumed as monodisperse [65], the analysis of DLS data with PdI-values above 0.1 by a cumulant fit can become unreliable. Hence, we used an alternative algorithm for data analysis instead, that is an exponential regularized fit in combination with non-negative least squares (NNLS) [65]. Using this method, a distribution of different particle size populations can be obtained. Our data revealed the presence of smaller (diameter 20–50 nm) and larger (200–1200 nm) particles in our bolasome sample after seven days of storage (Figure 4B). Hence, we assumed an instability of our **PC-C32(1,32C9)-PC**/soyPC vesicles under the given storage conditions. This is kind of remarkable, since the corresponding DOPC vesicles are stable in size over this time period. It is conceivable that the soyPC caused this stability issue. This observation is consistent with previous studies where the stability of lecithin vesicles has been investigated [66,67]. 

Despite the stability issue of the 1/4 **PC-C32(1,32C9)-PC**/soyPC mixture after seven days of storage, **PC-C32(1,32C9)-PC** was also miscible with natural occurring lipid mixtures such as soyPC shown by DSC and TEM investigations. In the next step, we wanted to discover if bolalipids incorporated in phospholipid vesicles can increase the integrity of the mixed vesicles (bolasomes). 

### 3.2. Integrity of Bolasomes 

To study the integrity of our mixed bolalipid/phospholipid vesicles (bolasomes) against small ions and in digestive media, three different compositions of bolasomes were analyzed, namely **PC-C32(1,32C6)-PC**/DOPC (1/4, *n*/*n*), **PC-C32(1,32C9)-PC**/DOPC (1/4, *n*/*n*), and **PC-C32(1,32C9)-PC**/ soyPC (1/4, *n*/*n*). The characterization of the first two compositions was published previously [53]. 

#### 3.2.1. Permeability of Bolasomes Against Small Ions: The Dithionite Assay 

It is well known that the cell membranes of some species of Archaea, which contain high amounts of membrane-spanning TELs, have a lower permeability against small ions such as protons compared to classical phospholipid membranes [68]. As a result, these organisms (thermoacido-philes) are able to survive at very low pH values. This low permeability against small ions is a desirable property of our bolasomes intended to use orally, which could result in the stability of bolasomes in the stomach environment. 

**Figure 5:**
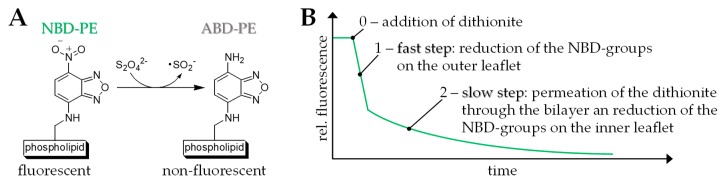
(**A**) Reduction of the green-fluorescent 1,2-dipalmitoyl-*sn*-glycero-3-phosphoethanolamine-*N*-(7-nitro-2,1,3-benzoxadiazol-4-yl) (ammonium salt) (NBD-PE) to the non-fluorescent 7-amino-2,1,3-benzoxadiazol-4-yl (ABD-PE) using dithionite. (**B**) Schematic representation of the two-step fluorescent decay using the dithionite assay. For details please refer to the text.

In order to study the permeability of our mixed bolalipid/phospholipid vesicles, we used a fluorescence assay established by McIntyre et al. [57]. This assay has been used by several research groups in the last decades to study the properties of model and cell membranes [57,69,70,71,72,73,74]. In this assay, the vesicles are labelled with 1 mol% of 1,2-dipalmitoyl-*sn*-glycero-3-phosphoethanolamine-*N*-(7-nitro-2,1,3-benzoxadiazol-4-yl) (ammonium salt) (NBD-PE)—a phospholipid that bears a fluores-cent moiety in the headgroup. The NBD-group can be reduced using different reducing agents such as dithionite (Figure 5A). By adding an excess of dithionite, the NBD-moieties are reduced to the non-fluorescent 7-amino-2,1,3-benzoxadiazol-4-yl (ABD) analogue and the fluorescent intensity of the vesicle suspension, which can be observed at an emission wavelength of 538 nm, decreases over time. This fluorescent decay is a two-step process (Figure 5B). First, the NBD-groups on the outer leaflet of the vesicles are reduced, which results in a fast decrease in the fluorescent intensity. Second, dithionite ions permeate the lipid bilayer and reduce the NBD-groups on the inner leaflet of the vesicles. The second decay is slower compared to the first one and the reduction rate is limited by the speed of the dithionite permeation. Moreover, the second step can be fitted by an exponential decay giving the rate constant *k*, which can be compared between different vesicle formulations. 

**Figure 6:**
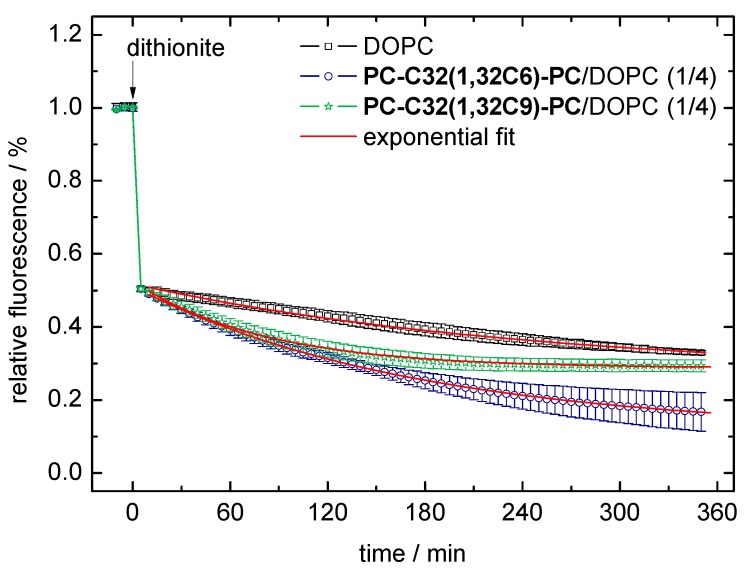
Data of the dithionite assay using 1,2-dioleoyl-*sn*-glycero-3-phosphocholine (DOPC) vesicles (black scattered data), **PC-C32(1,32C6)-PC**/DOPC (1/4, *n*/*n*; blue scattered data), and **PC-C32(1,32C9)-PC**/DOPC (1/4, *n*/*n*; green scattered data) bolasomes, respectively. Data points and error bars represent the mean and s.d. from three separate experiments. The red line corresponds to the exponential fit of the data points starting at *t* = 5 min.

With the use of this dithionite assay, we examined the permeability of pure DOPC vesicles and DOPC vesicles containing 20 mol% of **PC-C32(1,32C6)-PC** and **PC-C32(1,32C9)-PC**, respectively. The fluorescent decay data (scattered data) and the exponential fit (red line) of the second step in reduction of the NBD-groups are shown in Figure 6. From the exponential fit, we could obtain the rate constant *k*, which correlates to the velocity of the dithionite permeation, and the number of NBD-PE lipids in the inner leaflet of the vesicles. These data are summarized in Table 1. 

In all vesicle compositions investigated, the correlation between data and fit (*R*^2^) was greater than 0.99. This indicated that the diffusion (permeation) of the dithionite ions through the lipid bilayer can be described as an exponential process. However, it cannot be ruled out that during the long period of the measurement, a flip-flop of the NBD-PE lipids as well as reduced reactivity of the dithionite ions due to oxidation influenced the progression of the reaction [75]. 

By comparing the rate constants *k* taken from the exponential fit, we found that the permeation of the dithionite ions across the bilayer was roughly twice as fast for the bolalipid-modified vesicles compared to the unmodified DOPC vesicles: 5.8 × 10^−3^ min^−1^/7.1 × 10^−3^ min^−1^ *versus* 3.0 × 10^−3^ min^−1^ (Table 1). At first glance, this observation is somewhat unexpected since bolalipids incorporated in lipid bilayers should increase the stability of the bilayer and decrease, in turn, the permeability against small ions as found in Archaea [68]. But, if we take the rough surface of our bolasomes into account (compare Figure 3B and FFEM pictures in [53]), which could be interpreted as a clustering effect of the bolalipids [64], small ions such as dithionite or protons could cross the bilayer along these membrane defects where bolalipids and phospholipids come into contact. Nevertheless, these results again confirmed the incorporation of the bolalipids **PC-C32(1,32C6)-PC** and **PC-C32(1,32C9)-PC** into phospholipid membranes by maintaining the liposomal structure. 

A second parameter, which can be calculated from the exponential fit, is the number of NBD-PE lipids in the inner leaflet of the vesicles (Table 1). In theory, this value should be less than 50% due to the membrane curvature. However, we found values that were slightly larger than 50% (51.1–57.2%) because of the existence of some oligolamellar and/or multivesicular vesicles [53]. These additional “inner” layers contributed to a larger amount of inner NBD-PE lipids.

#### 3.2.2. Stability of Bolasomes in Different Digestive Media

Orally administered drug carriers have to overcome many obstacles until the optimum release point of their cargo. The first hurdle is the low pH value found in the stomach, pH 1–3 in the fasted state, in combination with the secretion of pepsin. Unmodified liposomes tend to lose their integrity in media with high proton concentrations, resulting in a premature release of cargo [17]. Later, the pH value rises to about 5–8 in the intestine and enzymes (for example trypsin) as well as bile salts are secreted. Bile salts are especially considered to be the most serious hurdle for lipid formulations, since the incorporation of bile salts into lipid membranes leads to the solubilization of vesicles into small mixed micelles [38,76,77,78]. 

**Figure 7:**
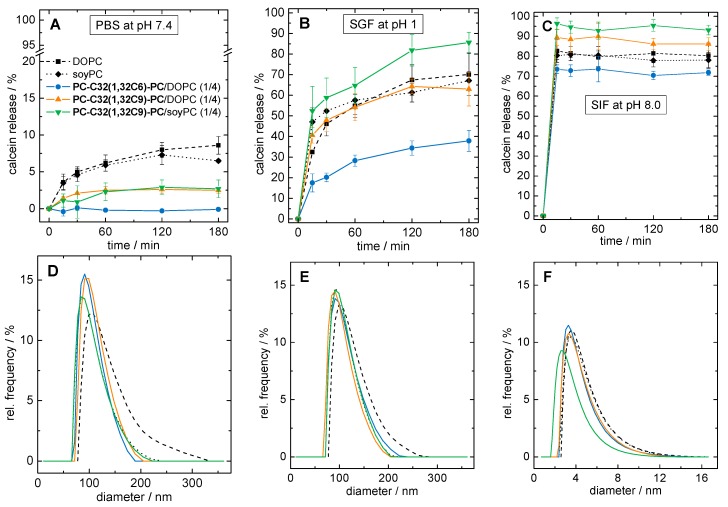
(**A**–**C**): Calcein release of different liposomal formulations during 3 h of incubation at 37 °C in (**A**) phosphate buffered saline (PBS) at pH = 7.4, (**B**) simulated gastric fluid (SGF) at = pH 1.0, and (**C**) simulated intestinal fluid (SIF) at pH = 8.0. (**D**–**F**): Volume-weighted particle size distribution of vesicle suspensions after 4 h of incubation at 37 °C in (**D**) PBS at pH = 7.4, (**E**) SGF at pH = 1.0, and (**F**) SIF at pH = 8.0. As liposomal formulations, pure DOPC (black squares, dashed line), pure phospha-tidylcholines from soy beans (soyPC) (black diamonds, dotted line), **PC-C32(1,32C6)-PC**/DOPC (1/4; blue data), **PC-C32(1,32C9)-PC**/DOPC (1/4; orange data), and **PC-C32(1,32C9)-PC**/soyPC (1/4; green data) were used.

We therefore tested the stability of our three bolasome compositions loaded with calcein in phosphate buffered saline at pH = 7.4 (PBS), SGF containing pepsin at pH = 1.0, and SIF containing trypsin and sodium taurocholate at pH = 8.0, respectively. We checked particle size, particle size distribution, and the release of calcein during the incubation at 37 °C. Vesicles of pure DOPC and soyPC were used as reference. The results of the calcein release assay are summarized in Figure 7A–C, whereas the particle size distributions after 4 h of incubation are shown in Figure 7D–F. 

First, we discuss the stability of our vesicles in PBS at pH = 7.4. After 3 h of incubation, 8.6 ± 1.2% and 6.5 ± 0.2% of the calcein was released from DOPC and soyPC vesicles, respectively (Figure 7A, black squares and diamonds). By contrast, the **PC-C32(1,32C6)-PC**/DOPC vesicles showed almost no release after 3 h (Figure 7A, blue circles) indicating a pronounced stabilization of the DOPC bilayer. In the case of both **PC-C32(1,32C9)-PC** modified vesicles, a reduction in calcein release of 60–70%, compared to unmodified DOPC and soyPC vesicles, could be obtained. The release of **PC-C32(1,32C9)-PC**/DOPC vesicles was found to be 2.5 ± 0.4% (Figure 7A, orange triangles), whereas the corresponding soyPC vesicles released 2.7 ± 1.2% of the calcein (Figure 7A, green triangles). After 4 h of incubation, all vesicles showed a monomodal particle size distribution with diameters ranging from 60–250 nm and a maximum of about 100 nm (Figure 7D). Hence, it is conceivable that calcein diffuses through an intact lipid bilayer in the case of both unmodified and modified vesicles. 

The situation changed when acidic conditions in combination with pepsin were used. During the incubation in SGF, 70.1 ± 10.3% and 67.1 ± 4.3% of calcein was released from DOPC and soyPC vesicles after 3 h of incubation, respectively (Figure 7B, black squares and diamonds). Again, DOPC vesicles modified with 20 mol% of **PC-C32(1,32C6)-PC** released only 37.9 ± 5.1% of calcein (Figure 7B, blue circles), which corresponds to a reduction of roughly 50%. Interestingly, the release from **PC-C32(1,32C9)-PC** doped vesicles remained unaffected—that is a 63.0 ± 8.2% release from **PC-C32(1,32C9)-PC**/DOPC vesicles (Figure 7B, orange triangles)—or even increased to 85.6 ± 4.9% in the case of the **PC-C32(1,32C9)-PC**/soyPC mixture (Figure 7B, green triangles). As found for the incubation in PBS, all vesicles showed an unchanged monomodal particle size distribution after 4 h of incubation (Figure 7E). It could therefore be concluded that—presumably due to the higher proton concentration in the SGF—a destabilization of the vesicle membrane occurred, which further promoted a release of the fluorescent dye. However, a complete lysis of the liposomes was not observed during the measurement time. The fact that the physicochemical properties of vesicles were only slightly affected by incubation in SGF and that the vesicles retained their size was also found in earlier studies [17,76]. The different effect of the bolalipid **PC-C32(1,32C9)-PC** on the release of calcein from DOPC and soyPC vesicles is kind of remarkable, since both unmodified vesicles showed a nearly identical release. It is known, that the calcein permeability through intact vesicle membranes depends on many factors such as the number and fluidity of the vesicle membranes, their bending elasticity, and their temperature [58]. Also, the osmolality of the surrounding medium can influence the calcein release. Nevertheless, the membrane-stabilizing properties of our bolalipid **PC-C32(1,32C9)-PC** in SGF seem not to work for every type of vesicle. 

Lastly, we checked the stability of our vesicles in SIF containing trypsin and bile salts. In fact, both unmodified vesicles as well as all bolasomes released over 70% of calcein within the first 10 min of incubation. The release after 3 h was found to be 80.3 ± 6.3% for DOPC vesicles (Figure 7C, black squares), 78.1 ± 3.9% for soyPC vesicles (Figure 7C, black diamonds), 71.8 ± 1.3% for **PC-C32(1,32C6)-PC**/DOPC vesicles (Figure 7C, blue circles), 86.1 ± 3.2% for **PC-C32(1,32C9)-PC**/DOPC vesicles (Figure 7C, orange triangles), and 93.1 ± 1.3% for **PC-C32(1,32C9)-PC**/soyPC vesicles (Figure 7C, green triangles). Particle size measurements after 4 h of incubation revealed the presence of very small particles with diameters between 2–12 nm for all samples (Figure 7F). Both, the rapid calcein release and the reduction in particle size could be explained by the solubilization of the vesicles by bile salts leading to the formation of small mixed micelles. It is obvious that bolalipids incorporated in the lipid bilayer cannot prevent the solubilization of the vesicles by the bile salts. The fact that only 70–95% of calcein was released during 3 h—instead of 100%—could be explained by the variable lamellarity of the different vesicles. It is known, that multilamellar vesicles are solubilized by a fast initial process of micelle formation followed by a slow step involving a successive “peeling-off” of the lamellar layers of the vesicles [79]. This “incomplete” release of calcein from vesicles incubated in media containing bile salts has been reported previously [76,80]. 

#### 3.2.3. Freeze-Drying and Re-Hydration of Bolasomes 

Instabilities of vesicles are usually related to oxidation processes, the hydrolysis of lipids, drug leakage, the formation of smaller aggregates, or vesicle fusion. All these issues result in an alteration of the in-vivo distribution, and the therapeutic efficiency of the encapsulated drug [9,81,82,83]. Because these phenomena are facilitated in the aqueous environment—and with a further perspective to later industrial production—the formulation of vesicles into a solid dosage form by freeze-drying is a promising approach to overcome these obstacles [84,85,86,87,88]. Since the removal of the aqueous environment can cause significant and sometimes irreversible alterations on the structural integrity of vesicles [89], the use of cryoprotectants is obligatory. These substances replace the water bound via hydrogen bonds to the surface of the vesicles, which results in a stabilization of the vesicle during freeze-drying [90]. As cryoprotectants, sugars such as mannitol, lactose, trehalose, or sucrose and amino acids such as proline or lysine are commonly used [88]. Lysine especially seems to be suitable for the stabilization of freeze-dried vesicles composed of natural unsaturated lipids [91]. Moreover, the use of trehalose as cryoprotectant results in an increased re-hydration efficiency of liposomes, compared to other sugars [59,88]. 

**Figure 8:**
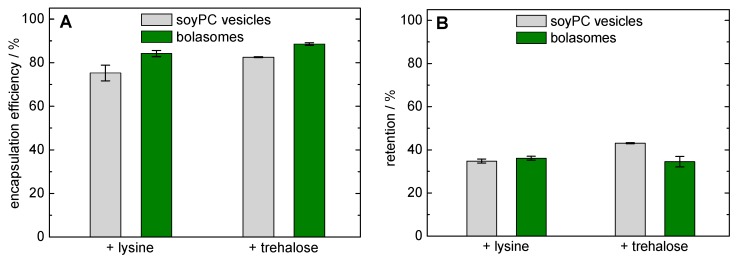
(**A**) Encapsulation efficiency (%) of calcein in unmodified soyPC vesicles (grey bars) and **PC-C32(1,32C9)-PC**/soyPC vesicles (1/4, *n*/*n*; green bars) prior freeze-drying using PBS including lysine (left-hand side) or trehalose (right-hand side). (**B**) Calcein retention (%) after freeze-drying and re-hydration of soyPC vesicles (grey bars) and **PC-C32(1,32C9)-PC**/soyPC vesicles (green bars) using lysine (left-hand side) or trehalose (right-hand side) as cryoprotectant.

For this reason, we used lysine at a concentration of 40 mM and trehalose at a concentration of 300 mM, respectively, as cryoprotectants for the encapsulation of calcein in **PC-C32(1,32C9)-PC**/ soyPC vesicles. Before the freeze-drying process, we determined the encapsulation efficiency of calcein in soyPC and **PC-C32(1,32C9)-PC**/soyPC vesicles (Figure 8A). Using lysine and trehalose, the bolalipid-doped vesicles showed a slightly higher encapsulation efficiency compared to unmodified soyPC vesicles (84.2 ± 1.4% and 88.5 ± 0.6% *versus* 75.3 ± 3.6% and 82.5 ± 0.2%,). Since both types of vesicles were not extruded for this experiment, a different lamellarity of the vesicles might also affect this result. 

In order to assess the stability of our vesicles during the lyophilization process, the retention of calcein was investigated. After freeze-drying with lysine as cryoprotectant, a calcein retention of 34.8 ± 0.9% was determined for soyPC vesicles and 36.1 ± 0.9% was found for the corresponding bolalipid-modified vesicles (Figure 8B, left-hand side). These values are comparable to values found the calcein retention using freeze-dried phospholipid vesicles and monosaccharides as cryoprotectants [92]. Hence, the use of bolalipid vesicles showed no advantage, but also no disadvantage, compared to classical unmodified vesicles regarding the stability during freeze-drying. With the use of trehalose as a cryoprotectant, the calcein retention was slightly increased to 43.1 ± 0.3% when soyPC vesicles were used. Here, the addition of 20 mol% **PC-C32(1,32C9)-PC** resulted in a slight decrease in calcein retention (34.5 ± 2.4%; Figure 8B, right-hand side). Therefore, the reasons remain unclear for now. Also the over-all lower calcein retention compared to other studies [59,88,92], when trehalose is used as cryoprotectant, should be clarified in the future. 

## 4. Conclusions 

In this study, we investigated three different bolalipid/phospholipid mixtures in order to discover whether bolalipids could increase the integrity of liposomes in gastrointestinal fluids. Firstly, we showed that **PC-C32(1,32C9)-PC**—one of the bolalipids used—is also miscible with soyPC, a prerequisite of the intended use. In the second part, we investigated the stability of **PC-C32(1,32C6)-PC**/DOPC, **PC-C32(1,32C9)-PC**/DOPC, and **PC-C32(1,32C9)-PC**/soyPC mixtures against small ions, in different digestive media, and during lyophilization. 

Although bolalipid-doped vesicles were roughly two times more permeable for small ions compared to unmodified DOPC vesicles, we could prove that the addition of bolalipids increased the stability in phosphate buffered saline, and in the case of **PC-C32(1,32C6)-PC**/DOPC, also in simulated gastric fluid. Unfortunately, all bolasome compositions were rapidly solubilized in simulated intestinal fluid within the first 10 min, and we did not observe any benefit of the bolasomes compared to unmodified vesicles with respect to the retention of encapsulated calcein during lyophilization.

Nevertheless, the artificial single-chain bolalipids **PC-C32(1,32C6)-PC** and **PC-C32(1,32C9)-PC** stabilized phospholipid membranes under certain physiological conditions. Especially the 1/4 **PC-C32(1,32C6)-PC**/DOPC mixture seems to be a very promising candidate as a novel vesicle-based oral drug delivery system. Further investigations including the use of different molar mixtures and the incorporation of peptide model drugs are part of ongoing research.

## Figures and Tables

**Table 1:** Data from the exponential fit of the second decay within the dithionite assay (Figure 6).

Vesicle Composition	Exponential Fit ^1^ $\left(y=a\cdot e^{k\cdot x}+c\right)$				Inner NBD Lipids (%) ^2^
	*a*	*k*	*c*	*R* ^2^	
DOPC	0.310	3.0 × 10^−3^	0.224	0.996	55.4
**PC-C32(1,32C6)-PC**/DOPC (1/4)	0.396	5.8 × 10^−3^	0.115	0.999	51.1
**PC-C32(1,32C9)-PC**/DOPC (1/4)	0.318	7.1 × 10^−3^	0.191	0.999	57.2

^1^ The fitting of data points starts at *t* = 5 min, that is 5 min after the addition of dithionite, to ensure complete reduction of the NBD-PE lipids on the outer leaflet of the vesicles. ^2^ This value equals *a* + *c*.
